# The Fabrication of Halogen-Doped FeWO_4_ Heterostructure Anchored over Graphene Oxide Nanosheets for the Sunlight-Driven Photocatalytic Degradation of Methylene Blue Dye

**DOI:** 10.3390/molecules28207022

**Published:** 2023-10-10

**Authors:** Muhammad Irfan, Noor Tahir, Muhammad Zahid, Saima Noreen, Muhammad Yaseen, Muhammad Shahbaz, Ghulam Mustafa, Rana Abdul Shakoor, Imran Shahid

**Affiliations:** 1 Department of Chemistry, University of Agriculture, Faisalabad 38040, Pakistan; mirfanjbd786@gmail.com (M.I.); noortahir17@yahoo.com (N.T.); saima_bashir03@yahoo.com (S.N.); 2 Department of Physics, University of Agriculture, Faisalabad 38040, Pakistan; 3 Punjab Institute of Nuclear Medicine, Faisalabad 38800, Pakistan; 4 Department of Chemistry, University of Okara, Okara 56300, Pakistan; 5 Center for Advanced Materials (CAM), Qatar University, Doha P.O. Box 2713, Qatar; 6 Environmental Science Center, Qatar University, Doha P.O. Box 2713, Qatar

**Keywords:** iodine doping, halogenation, methylene blue degradation, photo-Fenton, heterogeneous catalysis, wastewater treatment

## Abstract

Rapid industrialization and urbanization are the two significant issues causing environmental pollution. The polluted water from various industries contains refractory organic materials such as dyes. Heterogeneous photocatalysis using semiconductor metal oxides is an effective remediation technique for wastewater treatment. In this research, we used a co-precipitation-assisted hydrothermal method to synthesize a novel I-FeWO_4_/GO sunlight-active nanocomposite. Introducing dopant reductive iodine species improved the catalytic activity of FeWO_4_/GO. I^−^ ions improved the catalytic performance of H_2_O_2_ by doping into FeWO_4_/GO composite. Due to I^−^ doping and the introduction of graphene as a support medium, enhanced charge separation and transfer were observed, which is crucial for efficient heterogeneous surface reactions. Various techniques, like FTIR, SEM-EDX, XRD, and UV–Vis spectroscopy, were used to characterize composites. The Tauc plot method was used to calculate pristine and iodine-doped FeWO_4_/GO bandgap. Iodine doping reduced the bandgap from 2.8 eV to 2.6 eV. The degradation of methylene blue (MB) was evaluated by optimizing various parameters like catalyst concentration, oxidant dose, pH, and time. The optimum conditions for photocatalysts where maximum degradation occurred were pH = 7 for both FeWO_4_/GO and I-FeWO_4_/GO; oxidant dose = 9 mM and 7 mM for FeWO_4_/GO and I-FeWO_4_/GO; and catalyst concentration = 30 mg and 35 mg/100 mL for FeWO_4_/GO and I-FeWO_4_/GO; the optimum time was 120 min. Under these optimum conditions, FeWO_4_/GO and I-FeWO_4_/GO showed 92.0% and 97.0% degradation of MB dye.

## 1. Introduction

Water contamination continuously increases due to the nonbiodegradability of industrial and agricultural wastes, resulting in severe diseases in humans and aquatic organisms [1]. Drinking and the utilization of polluted water cause approximately 1.4 thousand human deaths worldwide [2]. Nowadays, dyes are used in the pharmaceutical, textile, leather, and bleaching industries and for coloring purposes [3]. Textile processing loses almost half the amount in water bodies. Due to solubility and photoresistance stability, dyes contaminate water and cause various diseases such as jaundice, nausea, and cyanosis. Water resources become detrimental due to the release of dyes into the drinking water [4]. Such hazardous pollutants require extraordinary efforts [5]. Various techniques have been used to remove wastewater contaminants, such as filtration, chlorination, adsorption, reverse osmosis, precipitation, coagulation, and ion exchange [6]. These techniques have various limitations, including by-product formation [7], as well as being time-consuming and inefficient [8,9].

Advanced oxidation technologies have received great attention due to their role in water disinfection and dye degradation. The advanced oxidation process is the most crucial method for degrading many inorganic and organic pollutants released from industrial waste [10]. Different types of AOPs are used for water remediation, such as heterogeneous photocatalysis, Fenton, sono-Fenton, and photo-Fenton [11]. Advanced oxidation processes (AOPs) for wastewater treatment and the elimination of obstinate sustainable particulates have also been rapidly developed. Under ambient conditions, green technology AOPs utilize solar energy and help to remove hazardous materials from water.

In AOPs, reactive oxygen species (ROS) are generated, which degrade organic pollutants more effectively. AOPs have advantages owing to the formation of free radicals resulting from chemical reactions. During degradation, oxidizing agents such as charge carriers, superoxide radicals, and hydroxyl radicals effectively turn pollutants into harmless compounds. Various photocatalysts, such as metal ferrites, metal halides, and metal tungstates, have advantages in heterogeneous photocatalysis. Various limitations such as fast recombination of charge carriers, a lack of cost-effectiveness, and low surface area can hinder the degradation process [12]. The efficiency is improved using adsorbents that provide a surface for photocatalyst immobilization.

Among AOPs, the heterogeneous Fenton process is the most efficient and renewable. Due to its ability to degrade organic pollutants, the heterogeneous Fenton reaction, which uses Cu and Fe compounds and H_2_O_2_, has been intensively developed [13,14]. The consumption of H_2_O_2_ increases due to the low efficiency of the heterogeneous Fenton process. To overcome these limitations, composites of reductive metals like Fe, Mn, Cu, Ni, and Zn are used to activate H_2_O_2_, which improves the degradation of contaminants [15,16]. Reductive species such as I^−^ have good reducibility and therefore are used for improving the heterogeneous Fenton process. I^−^ is a more potent catalyst than a Fenton catalyst like Fe^2+^.

Metal tungstates are ternary compounds that show excellent photocatalytic activity due to narrow bandgap and crystalline structure, resulting in the efficient utilization of sunlight and thus enhanced photoresponse [17]. The recombination time of photoexcited species is mainly reduced due to the narrow bandgap of ternary compounds. Some modifications are needed to improve the catalytic activity of metal tungstates. In the past decade, the photocatalytic activity of semiconductors was enhanced through several modification methods [18]. The coupling of metal tungstate with semiconductors with narrow bandgaps or transition metal doping enhances the photocatalytic activity of metal tungstate. The recombination time of charge carrier species is increased by providing a new energy level for charge carriers at which the trapping of e^−^ occurs through heterojunction formation. Photoexcited electrons are trapped at this new energy level, and doping enhances ternary compounds’ activity responsiveness toward the visible region of ternary compounds, by introducing foreign ions, changing nanocomposite morphologies and changing the bandgap. The new energy band is formed above the valence band of the host due to the empty “d” orbitals of nonmetals. Due to this energy level, the photocatalyst shows degradation activity in the visible region through redshift in the bandgap [19]. 

The supporting material, graphene oxide (GO), increases the surface area due to sp^2^ hybridization. The recombination time of holes and electrons increases by electrons shuttling between active co-catalyst sites and the photocatalyst, enhancing catalytic activity. Additionally, the photoresponse is enhanced through the adsorption of pollutants on the surface of GO. Tungstate composite with GO enhances the catalytic activity. P-type FeWO_4_, with a bandgap of 2.0 eV, belongs to the wolframite family, which shows excellent photocatalytic activity due to various optical, electronic, and ferromagnetic properties [20].

In the present work, a novel I-FeWO_4_/GO (I-FWGO) photocatalyst was synthesized and effectively used for methylene blue (MB) degradation under sunlight. The iodine doping of FeWO_4_ enhanced the degradation efficiency. The iodine-doped FeWO_4_ was hydrothermally treated with GO to form the doped hybrid composite. The novel I-doped FeWO_4_/GO was characterized using FTIR, XRD, and SEM-EDX. Dye concentration and bandgap were monitored using UV–Vis spectroscopy. The dye degradation was assessed and optimized using activity parameters like time, oxidant dose, pH, and catalyst concentration. Iodine doping enhanced the photocatalytic activity of the composite in the visible region. 

## 2. Results and Discussion

### 2.1. FTIR Analysis

**Figure 1:**
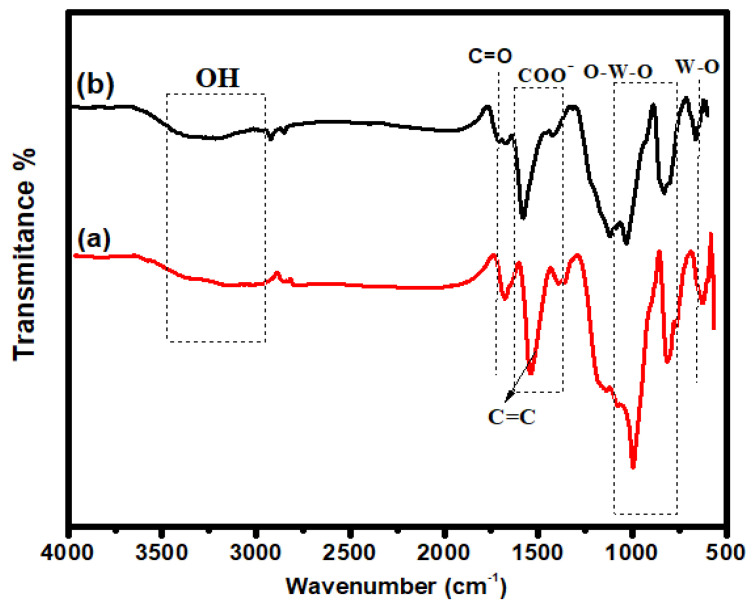
FTIR spectra: (**a**) FeWO_4_/GO; (**b**) I-FeWO_4_/GO.

The most important approach for finding composite functional groups is FTIR. FTIR provides information about the bonding that is present in composite materials. The characteristic peaks of undoped and iodine-doped iron tungstate/GO are shown in Figure 1. The broad absorption peaks around 3614 cm^−1^ and 3007 cm^−1^ are attributed to small -OH groups that were hydrothermally bonded to composites, indicating the presence of H_2_O on the surface [21]. The typical elongated band at 567 cm^−1^ is related to bending vibrations due to Fe-O. The characteristic peaks of GO are shown around 1410 cm^−1^, 1581 cm^−1^, and 1711 cm^−1^, which are attributed to COO^−^, C=C, and C=O stretching vibrations, respectively [22]. In both composites, two characteristic peaks correspond to W-O stretching and O-W-O stretching peaks at 832 cm^−1^ and 1034 cm^−1^ [23]. In I-FWGO, the stretching peaks of O-W-O and F-O-W shifted due to the iodine doping into the Fe-O lattice [24,25]. The peak intensity in the iodine-doped composite was lower than in the undoped counterpart and pristine FeWO_4_, confirming the successful insertion of dopant species in the host material [23]. 

### 2.2. XRD Analysis

**Figure 2:**
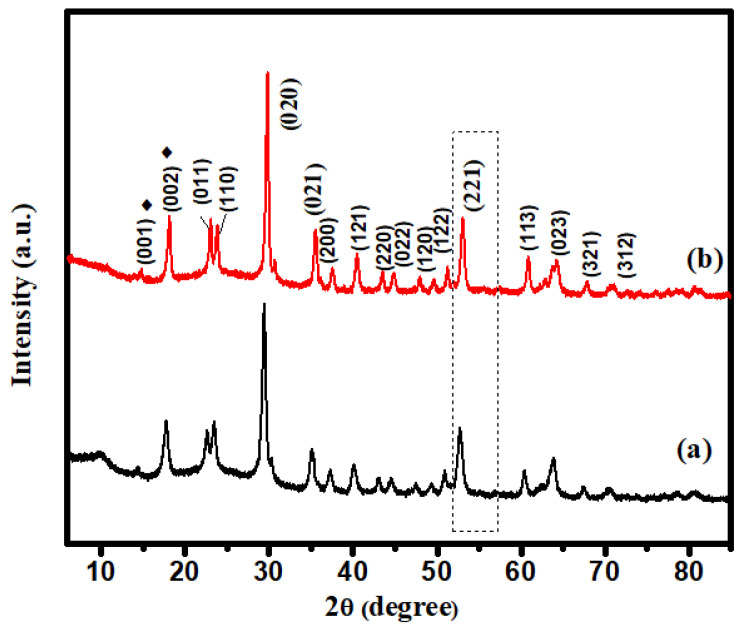
XRD spectra: (**a**) FeWO_4_/GO; (**b**) I-FeWO_4_/GO.

XRD may be used to determine the crystalline size of nanocrystals. Figure 2 depicts the XRD pattern of I-FWGO and FeWO4/GO. In XRD spectra, diffraction peaks were observed at 2θ = 24.7°, 25.5°, 31.4°, 32.5°, 37.3°, 39.3°, 42.2°, 49.6°, 51.3°, 52.8°, 54.7°, 62.4°, and 66.0°, which were indexed to (011), (110), (020), (021), (200), (121), (022), (220), (122), (202), (032) and (312) crystal planes of I-doped FeWO_4_/GO and FeWO_4_/GO, respectively (JCPDS No. 46-1446) [26].

The high intensities of the I-FWGO peak demonstrated the influence of iodine doping on the crystal structure. Iodine doping led to a shift in the peak toward a higher 2θ value, indicating that I^−^ substituted O^2−^ and increased the interlayer distance [27]. Crystal growth was restricted by the insertion of iodine, which blocked the grain boundary. Furthurmore, a slight shift in peak (221) was observed due to iodine doping [27]. The prominent peaks at 2θ = 13.6°, with d-spacings of 0.38 nm and 2θ = 17.1° in XRD patterns of both composites, are attributed to the (001) and (002) planes of graphene oxide, thus confirming its synthesis. The sharp peak at 2θ = 18.1°, corresponding to the (002) plane, might be attributed to restacked graphene sheets in the short-range order [28].

The Scherrer equation was used for crystalline size calculation, which is expressed in Equation (1).

(1) $$D=\frac{0.9\lambda }{\beta \cos\theta }$$

where the crystal’s crystalline size is “D”, the Scherrer constant “K” value is “0.94”, the wavelength “λ” of the X-ray source is 0.154 nm, the diffraction angle is “θ”, and the entire width of the half maximum is “β.” The crystalline sizes of I-FeWO_4_/GO and FeWO_4_/GO were 16.79 nm and 23.9 nm, respectively.

### 2.3. SEM-EDX

**Figure 3:**
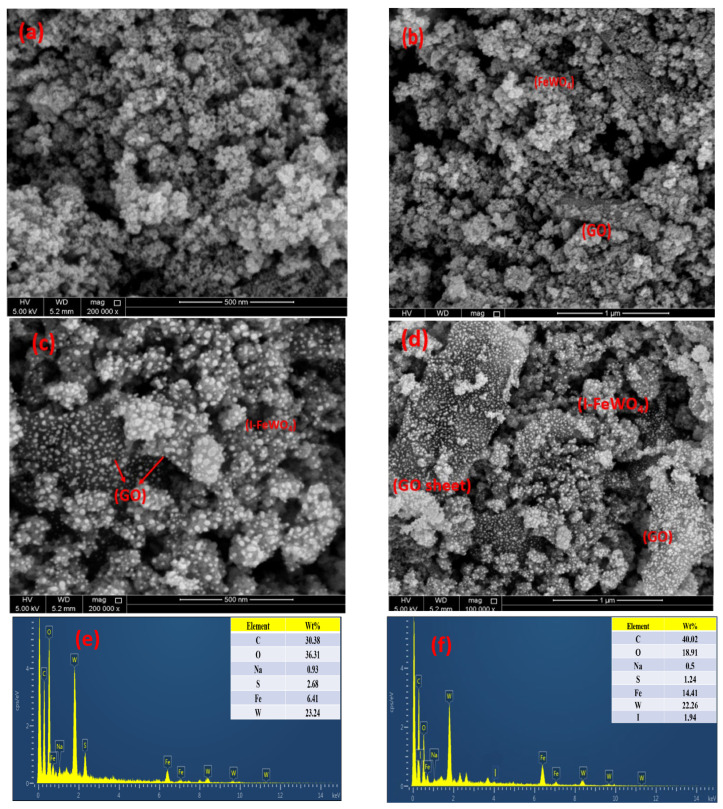
SEM images of FeWO_4_-GO (**a**,**b**) and I-FeWO_4_/GO (**c**,**d**) and EDX images of (**e**) FeWO_4_−GO and (**f**) I-FeWO_4_/GO.

SEM characterizes the morphology and morphological changes at various resolutions, as shown in Figure 3. It can be clearly seen that the spherical nanoflake-like structure of FeWO_4_ is well dispersed over small sheets of graphene oxide. Nanoparticle aggregation occurs due to the irregular edges of FeWO_4_/GO. The SEM shows spherical-shaped particles of I-FeWO_4_/GO with less agglomeration and uniformity of particles over small scattered sheets of GO. This uniformity and less aggregation result from surface area enhancement due to iodine doping [24]. The interaction of nonmetal ion species with metal tungstate forms interstitial spaces in metal oxide lattice. This ultimately leads to a faster nucleation rate and controlled growth kinetics, enhancing surface area [29]. The negatively charged iodide adsorbed on the composite surface, and as a result, the dispersion of nanoparticles increased in I-FeWO_4_/GO compared with FeWO_4_/GO. The iodine doping can be easily observed in SEM images of doped nanocomposite as compared to its undoped counterpart. The particle size of nanoparticles was computed using ImageJ v.1 software. The average particle size of FeWO_4_/GO and I-FeWO_4_/GO were 33 nm and 28.5 nm, respectively.

The elemental analysis of the prepared FeWO_4_/GO and I-FWGO was carried out through EDX, and the results are shown in Figure 3. Fe, W, O, and C show evidence of composite preparation with respective weight percentages in the inset. The iodine-doped FeWO_4_/GO nanocomposite shows the presence of iodine and the successful incorporation of iodine in the host lattice [30]. 

### 2.4. Optical Study of FeWO_4_/GO and I-FeWO_4_/GO

The bandgap and optical properties of novel I-FeWO_4_ and FeWO_4_ were measured by observing UV–Vis spectra ranging from 200 to 800 nm. The Tauc plot method was used to determine the absorption edges of I-FeWO_4_ and FeWO_4_ in the visible region. The formation of heterojunction with GO and iodine doping improved the absorption ability of I-doped FeWO_4_/GO. It enhanced the degradation activity by introducing new energy levels where electrons were quickly excited, and recombination was inhibited. The bandgap of the catalyst was calculated using Equation (2) as shown below:

(2) $${\left(\alpha hv\right)}^{2}=B\left(hv-Eg\right)$$

where *α*, *Eg*, *v*, and *h* represent the absorption coefficient, energy gap, light frequency, and proportionality constant. The plot between (αhv)^2^ and *hv* was used for determining the bandgap.

**Figure 4:**
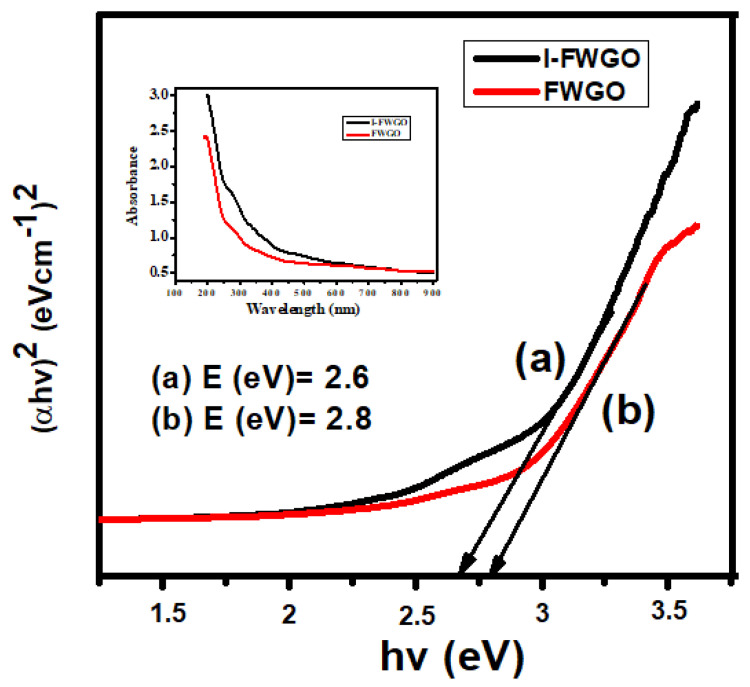
Bandgap of (**a**) I-FeWO_4_/GO and (**b**) FeWO_4_/GO.

In this composite, the bandgap of FeWO_4_/GO was 2.8 eV, which is larger than the bandgap of I-FWGO (2.6 eV), as shown in Figure 4. Iodine was present at the interstice spaces of the FeWO_4_/GO composite. The doped composite’s bandgap was reduced due to reactive iodine species in the interstitial matrix of FeWO_4_/GO, thereby enhancing the surface area. The photocatalytic activity of I-FWGO was more significant than FeWO_4_/GO due to the easy excitation of electrons in the conduction band and the suppression of the charge carrier’s recombination.

## 3. Operating Parameters

Photocatalytic activities of composites were studied using several parameters, including catalyst concentration, pH, amount of H_2_O_2_, and irradiation time.

### 3.1. pH Effect

**Figure 5:**
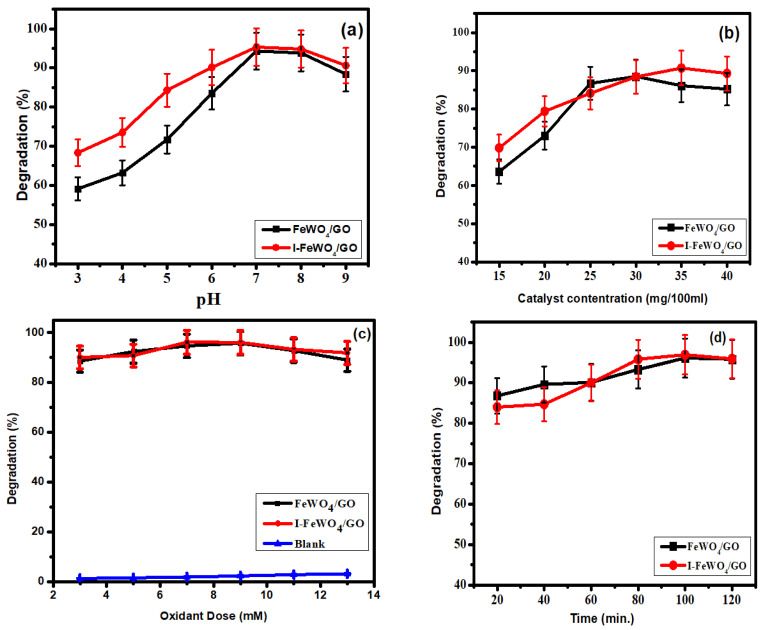
Optimized parameters: (**a**) pH; (**b**) catalyst load; (**c**) oxidant dose (H_2_O_2_); (**d**) time.

The pH value generally has an explicit influence on the photocatalytic degradation of hazardous dyes, and the breakdown performance is usually connected to the number of hydroxyl radicals (OH^•^) present in the medium, which significantly boosts the photodegradation efficiency in high-pH solutions. Additionally, the surface properties of photocatalysts play an essential role in the photodegradation of MB dye, which depends upon the pH of solutions. The influence of pH on the photocatalytic degradation of MB catalyzed by FWGO and I-FWGO nanocomposites is shown in Figure 5a. The degradation activity of the photocatalyst was changed under a broad range of pH (3–9) while preserving other variables unaltered (25 mg catalyst dosage and visible irradiation). The lowest decomposition performance was observed at the lowest pH value (pH = 3), with 58.0% and 68.0% of MB degraded using FWGO and I-FWGO after 100 min, respectively. However, increasing the pH to 7 resulted in the degradation of 94.3% and 95.3% of the MB dye in under 80 min using FWGO and I-FWGO respectively, due to the formation of many hydroxyl radicals [31]. Furthermore, dye adsorption on the catalytic surface increased with pH as MB dye has a positive charge and therefore is strongly adsorbed on negatively charged photocatalysts [29].

The pH is also depend on the point of zero charge, which is the threshold at which the surface of the photocatalyst has no charge. The photocatalyst has a negative charge on its surface if the pH is more than the point of zero charge. In the present study, the point of zero charge was 6.22 MB, and a cationic dye became readily adsorbed on the catalyst surface. Following thet, a reduction in photocatalytic activity was observed [32].

### 3.2. Influence of Catalyst Concentration on Photodegradation

The most critical parameter for the determination of photocatalytic activity is catalyst dose. The adsorption of dyes on catalyst surfaces plays an essential role in degradation. In this study, we used various catalyst concentrations (15–40 mg/100 mL dye solution) to determine the photocatalytic activity. The optimum concentrations for FWGO and I-FWGO were 30 mg and 35 mg respectively, at which maximum degradation rates of about 88.5% and 90.7% occurred under sunlight. FeWO_4_ showed about 96.8% methyl blue degradation under UV light after 120 min (Figure 5b). 

Initially, the degradation of MB increased sequentially with catalyst dose as the surface area of the catalyst was enhanced. More significant adsorption led to a higher rate of degradation. When the catalyst dose exceeded the optimum value, agglomeration occurred, and as a result, the surface area was reduced, and hence, the degradation of the dye decreased [21]. 

In this work, the heterojunction formation of FeWO_4_ with graphene oxide and iodine doping improved the recombination rate of photogenerated e^−^ and h^+^ by prolonging their suppression. Photoexcited electrons transfer from the conduction band (CB) of FeWO_4_ toward the CB of GO. In contrast, h^+^ transfers from the valence band (VB) of GO toward the VB of FeWO_4_, and hence, the chance of recombination decreases, and degradation activity is enhanced. Iodine doping also enhanced the catalytic activity by improving the surface area of the catalyst. Iodide oxide converts into iodine and then into iodate at low pH. Due to these ions, the surface of the catalyst becomes negatively charged. IO^3−^ is a potent oxidizing agent like peroxide [33].

### 3.3. Oxidant Dose

Under the following conditions, the oxidant (H_2_O_2_) amount was optimized: MB solution (30 ppm); pH = 7; and catalyst dose = 30 mg/100 mL for FeWO_4_/GO and 35 mg/100 mL for I-FWGO. The degradation of MB increased successively with oxidant dose (Figure 5c). The optimum oxidant value was 7 mM for I-doped FeWO_4_/GO and 9 mM for FeWO_4_/GO. The reduction of H_2_O_2_ to OH^•^ occurs by accepting electrons [34]. In photodegradation, the MB dye is effectively degraded by using OH^•^ radicals. Furthermore, OH^•^ radicals capture photoexcited electrons from the conduction band, and as a result, the recombination time increases.

$$H_{2}O_{2}+e_{CB}^{-}\to HO^{-}+HO^{•}$$

$$H_{2}O_{2}+O_{2}^{•-}\to HO^{-}+HO^{•}+O_{2}$$

Increasing the amount of H_2_O_2_ over the optimum value limits the degradation efficiency because an oxidant quenches the OH^•^ radical [35,36,37]. In some cases, the large amount of hydrogen peroxide generates hydrogen peroxide that is less reactive than hydroxyl radicals [21].

$$H_{2}O_{2}+HO^{•}\to H_{2}O+HO_{2}^{•}$$

$$HO_{2}^{•}+HO^{•}\to H_{2}O+O_{2}$$

Moreover, a reaction occurs between photogenerated holes and an excess amount of the oxidant, producing oxygen and proton. As a result, fewer $HO^{•}$ radicals are generated, resulting in less degradation. Moreover, in the absence of the oxidant, the catalyst does not exhibit any significant photocatalytic activity.

$$H_{2}O_{2}+2h_{VB}^{+}\to O_{2}+2H^{+}$$

### 3.4. Irradiation Time

**Figure 6:**
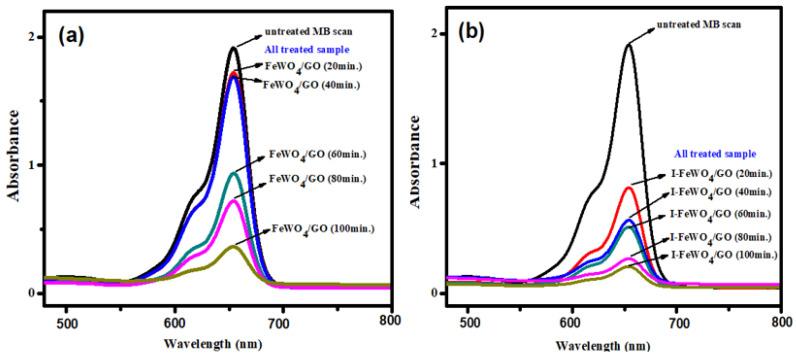
MB UV–Vis spectral changes during degradation over time using (**a**) FeWO_4_/GO and (**b**) I-FeWO_4_/GO.

The irradiation time is the most crucial parameter for analyzing the degradation activities of the photocatalyst. All three optimized parameters were constant for time optimization. Degradation was retarded at the start of the reaction due to the formation of intermediate species that required enough time for degradation. However, it climbed over time until it reached 120 min (Figure 5d). The FWGO and I-FWGO photocatalysts exhibited 92.1% and 97% degradation after 120 min. The UV–Vis scans of dye degradation over time are demonstrated in Figure 6, showing the degradation of MB dye by both doped and undoped nanocomposites over time.

### 3.5. Reaction Kinetics

**Figure 7:**
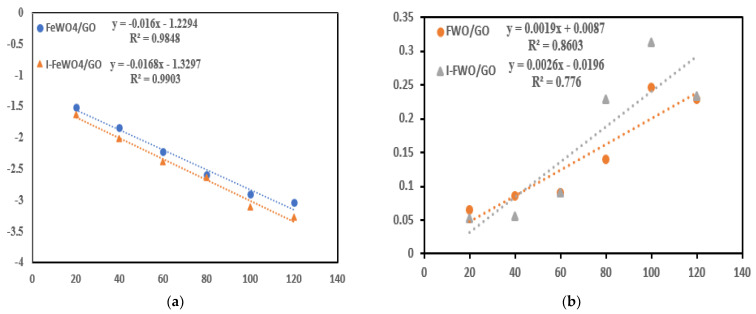
Reaction kinetics: (**a**) first-order kinetics; (**b**) second-order kinetics of FeWO_4_/GO and I-FeWO_4_/GO.

For the quantitative study of MB, the pseudo-first- and pseudo-second-order kinetic models were used. When the pollutant concentration is in the millimolar (mM) range, Equations (3) and (4) are used for catalytic experiments. The equations for first- and second-order kinetic models are as follows:

(3) $$\ln\frac{Ct}{Co}=-K_{1}t$$

(4) $$\frac{1}{Ct}-\frac{1}{Co}=K_{2}t$$

where *C_o_* is the initial dye concentration and *C_t_* is the cncentration of MB at a time “*t*”. For first- and second-order reaction kinetics, a linear response was observed from the plot of time (T) versus “ln (*C_t_*/*C_o_*)” and “*1*/*Ct*−1/*Co*”. The “R^2^” and “k” values are shown in Table 1. The values of “R^2^” and “k” were more significant for I-I-FeWO_4_/GO, indicating that iodine doping improved the catalytic activity (Figure 7).

### 3.6. Degradation Using UV Irradiation

The photocatalytic activity of the prepared composite was also investigated under ultraviolet light, keeping the experimental conditions obtained from batch studies constant. Using UV light, the catalytic activity of novel I-FeWO_4_/GO was analyzed by measuring absorbance using a spectrophotometer. Under optimized conditions, I-FeWO_4_/GO exhibited 70.8% degradation after 2 h, which shows that iodine composites are adequate sunlight-activated catalysts for the degradation of pollutants.

### 3.7. Reusability

**Figure 8:**
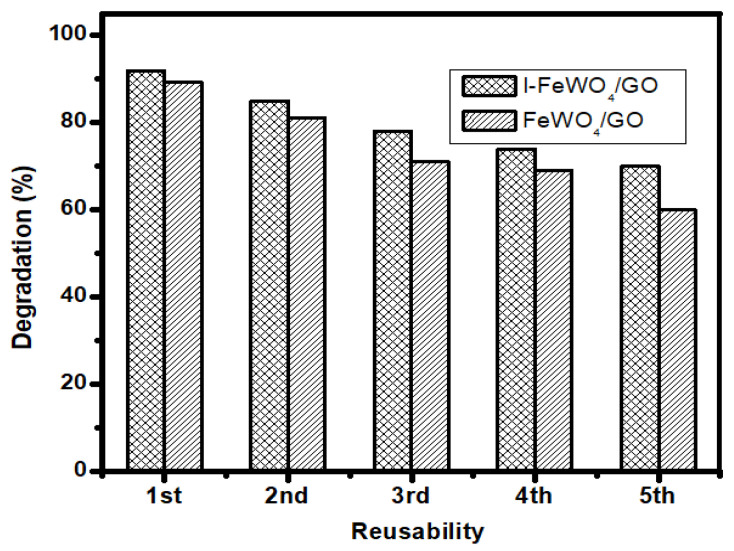
The reusability study using I-FeWO_4_/GO and FeWO_4_/GO.

The reusability experiment is significant for evaluating the economic feasibility of catalysts under various conditions. The composite’s stability was tested using a reusability trial test under optimized settings for up to five consecutive runs. All the optimized conditions from batch studies for the degradation of MB were kept constant. The sample was washed and dried five times and used for catalyst degradation. After five consecutive runs, the efficiency decreased from 97% to 75% using I-FeWO_4_/GO, as shown in Figure 8. 

### 3.8. Radical Scavenging Experiments and the Proposed Mechanism

**Figure 9:**
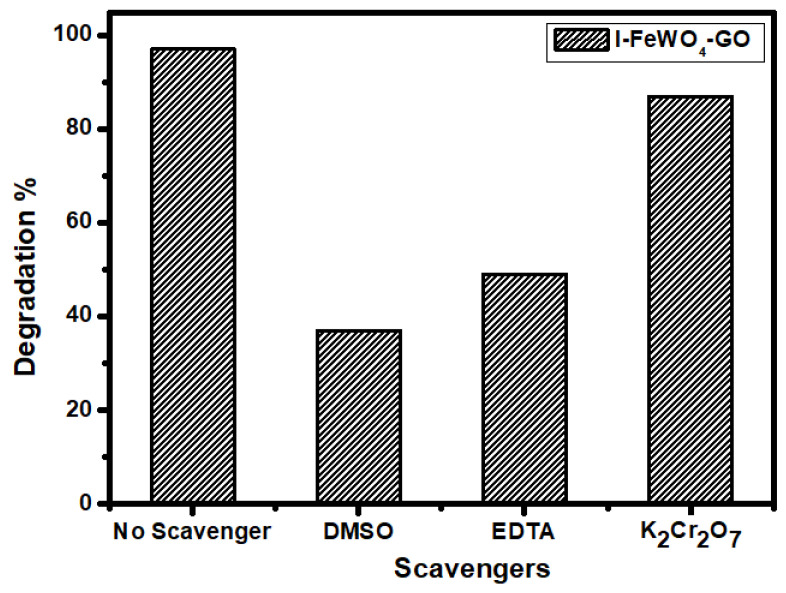
Radical scavenger effect of radical trapping scavengers.

Reactive species such as electron (e^−^), hole (h^+^), and hydroxyl radicals ($HO^{•}$) play a key role in the photocatalytic activity. The efficiency of the radicals involved in the degradation process can be investigated by using radical scavenging species. For this purpose, 5 mM radical scavengers were used. The radical scavenger K_2_Cr_2_O_7_ (potassium dichromate) was used for e^−^, EDTA (ethylenediaminetetraacetate) was used for h^+^, and DMSO (dimethyl sulfoxide) was used for $HO^{•}$ as shown in Figure 9. After adding a precise number of these scavengers under optimal working conditions, the dye solution with the catalyst was exposed to sunlight. The results show a drastic reduction in dye solution degradation using DMSO. The degradation was decreased from 97% to 37% by using DMSO for I-I-FeWO_4_/GO. Similarly, the contribution of electrons and hole scavengers also reduced the degradation.

#### The Proposed Mechanism of Degradation

**Figure 10:**
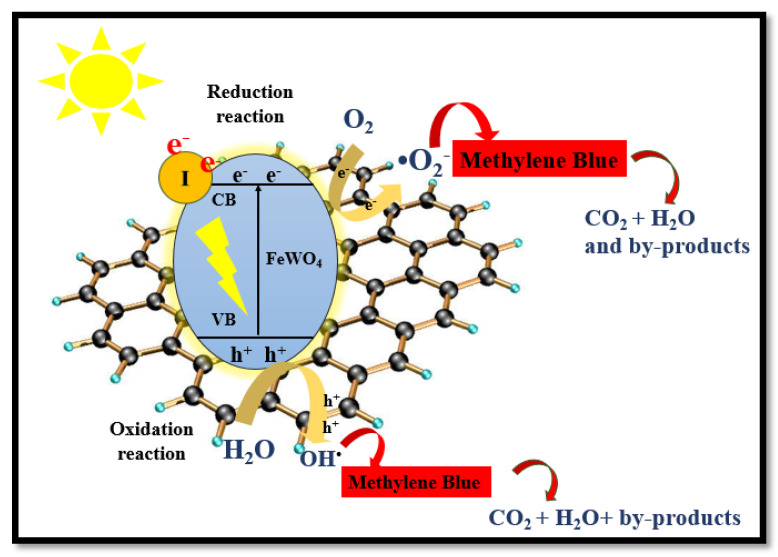
The proposed mechanism for the catalytic degradation of MB using I-FeWO_4_/GO.

Electrons and holes are the reactive oxygen species that initiate the degradation process. When sunlight falls on the I-FeWO_4_/GO surface, the electrons are rapidly excited from the VB toward the CB due to iodine doping, which reduces the bandgap of FeWO_4_. These photoexcited electrons move toward the CB of graphene oxide, and holes (h^+^) remain in the VB of FeWO_4_. In graphene oxide, h^+^ moves toward the VB of FeWO_4_. Fe^2+^ ions react with H_2_O_2_ and thus generate hydroxyl radicals and change into Fe^3+^ ions. The recombination time of the charge carrier is enhanced by receiving electrons from the conduction band by Fe^3+^. Therefore, the generation of photo-Fenton reagents leads to a high degradation rate at neutral pH. This photogenerated h^+^ reacts with H_2_O_2_, thus producing hydroxyl radicals. Additionally, many protons are present, which can react with $O^{•}$ and generate $OH^{{}^{\circ}}$, which is used for degradation. The electron (e^−^) in the CB oxidizes H_2_O_2_ to generate $OH^{•}$ as swn in Figure 10. The mechanism is as follows:

$$I-FeWO_{4}\left(h^{+}+e^{-}\right)/GO\left(e^{-}+h^{+}\right)\overset{hv}{\to }I-FeWO_{4}\left(h^{+}\right)+GO\left(e^{-}\right)\;$$

$$I-FeWO_{4}\left(h^{+}\right)+H_{2}O_{2}\to I-FeWO_{4}+H^{+}+OH^{•}$$

$$GO\left(e^{-}\right)+O_{2}\to GO+O_{2}^{•}$$

$$OH^{•}+OH^{•}\to H_{2}O+O^{•}$$

$$H^{+}+O^{•}\to OH^{•}$$

$$H_{2}O_{2}+O^{•}\to OH^{•}+OH^{-}+O_{2}$$

$$Fe^{2+}+H_{2}O_{2}\to Fe^{3+}+OH^{-}+OH^{•}$$

$$Fe^{3+}+H_{2}O_{2}\;\to Fe^{2+}+HOO^{•}+H^{+}$$

$$Fe^{3+}+H_{2}O\overset{hv}{\to }Fe^{2+}+OH^{•}+H^{+}$$

$$OH^{•}+MB\to Intermediate\to Degraded\;Products$$

### 3.9. The Optimization of Interacting Parameters Using Response Surface Methodology (RSM)

The RSM method was used to determine the effects of the variables on the methylene blue degradation. The response was predicted using the second-order polynomials given below.

(5) $$Y=\beta_{0}+\sum_{i=1}^{k}\beta_{i}x_{i}+\sum_{i=1}^{k}\beta_{\mathrm{ii}}x_{i}^{2}+\sum_{i=1}^{k}\sum_{i\neq j=1}^{k}\beta_{\mathrm{ij}}x_{i}x_{j}+\in$$

where the linear factor coefficient is “β_i_”; the % degradation of MB is indicated by “Y”; the variables of “j” and “i” are “xj” and “xi”; the intercept term is “β_0_”; the quadratic factor and the interaction factor are indicated by “β_ii_” and “β_ij_”; and “k” and “ε” represent the number of factors and the random error.

In Table 2, the results obtained using the Design-Expert v.1 software are shown. A regression model was developed as follows:

(6) $$Y\;=+95.20-0.47*A-0.78*B+0.058*C+3.30*D-7.92*A*B+1.55*A*C-1.42*A*D-\;6.50*B*C-0.20*B*D-3.40*C*D-13.49*A^{2}-13.58*B^{2}-3.50*C^{2}-3.12*D^{2}$$

where the MB degradation is represented by “Y’, and pH, oxidant dose (mM), catalyst load (mg/100 mL), and time (mint.) are represented by “A”, “C”, “B”, and “D”, respectively. The final Equation (6) shows the “linear” (A, B, C, and D), “interaction” (AB, AD, AC, BD, BC, and CD), and “quadratic” (A^2^, B^2^, C^2^, and D^2^) effects.

By using ANOVA, the validation of the model was investigated. The significance of the model was assessed by using the “F” value. The interaction of independent and dependent variables was investigated by using the R^2^ value.

#### Optimization through Response Surface Methodology for Iodine-Doped Iron Tungstate/Graphene Oxide (I-FeWO_4_/GO)

The antagonistic effects of pH and dye degradation are shown by negative signs. When the pH of the solution increased, the degradation % increased. At the same time, “B,” “C,” and “D” have + ve-signs, which show a synergistic effect on degradation. The dye degradation increased by increasing the oxidant dose, catalyst load, and time. 

**Figure 11:**
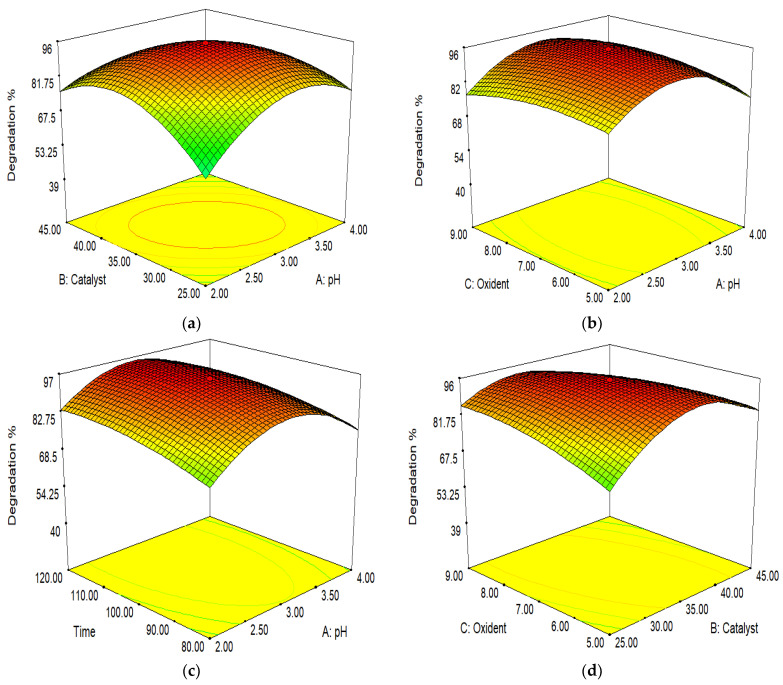

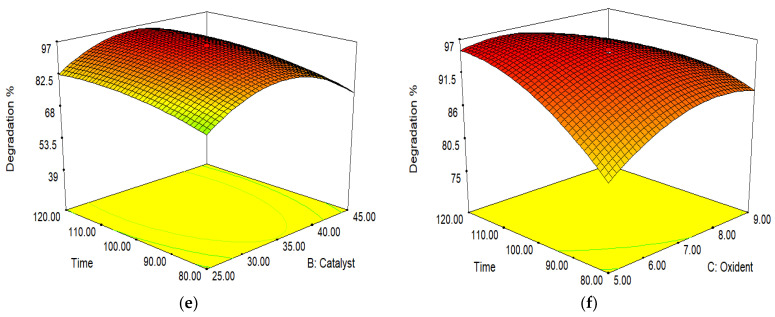
Interactions of (**a**) pH and catalyst load; (**b**) pH and oxidant dose; (**c**) pH and time; (**d**) oxidant dose and catalyst load; (**e**) time and catalyst load; and (**f**) time and oxidant dose.

The ionic form and surface charge were immediately affected by changing the pH of the solution. At pH 6.22, I-FeWO_4_/GO had zero charge on the surface. The mutual interaction between the catalyst concentration and pH is illustrated in Figure 11a. The catalyst concentration was set at 20–30 mg/100 mL, and pH was adjusted to 2–4. The degradation increased by increasing the pH of the solution to 7 due to the more significant interaction of MB with the catalyst. The degradation increased by increasing the catalyst concentration up to 25 mg/100 mL. The aggregation of the catalyst occurred when a high amount of catalyst was used; hence, a reduction in dye degradation was observed.

The pH level and time play an important role in the catalytic activity. Initially, the intermediate products formed require enough time for complete mineralization. Hence, the degradation of dye increases with time. The interaction between the oxidant dose and pH is depicted in Figure 11b. The oxidant dose (H_2_O_2_) plays a significant role in degradation. The oxidant dose ranged from 5 to 9 mM. At 7 mM, maximum degradation occurred due to the formation and interaction of $OH^{{}^{\circ}}$ with dye; a further increase would reduce the catalytic activity due to the reduction in $OH^{{}^{\circ}}$ [38]. 

The combined effect of the catalyst concentration and oxidant dose is shown in Figure 11d. Increasing the oxidant dose to 7 mM enhanced the photocatalytic activity for I-FeWO_4_/GO. The degradation of the dye remains constant if the amount of catalyst is increased, while the H_2_O_2_ concentration is low [39].

The interaction of the time parameter and the catalyst load is shown in Figure 11e. The catalytic activity was enhanced by increasing the concentration of the catalyst due to many active sites. If the catalyst concentration increased too much, degradation would decrease due to the agglomeration of the catalyst; hence, the surface area would be reduced. Initially, the degradation rate was low due to the production of resistant species. These intermediate products radially degraded with time [40].

## 4. Experimental Procedures

### 4.1. Materials and Reagents

In this study, an iodine-doped nanocomposite coupled with GO was prepared through a simple co-precipitation-assisted hydrothermal method. Analytical-grade reagents and chemicals were used for the synthesis of materials. Ammonium iron (II) sulfate ((NH_4_)_2_SO_4_·FeSO_4_·6H_2_O) (99%), sodium tungstate dihydrate (Na_2_WO_4_·2H_2_O) (97%), ethanol (95.6%), and potassium iodide (KI) were obtained from UNI-CHEM. Sodium nitrate (NaNO_3_), potassium permanganate (KMnO_4_), sulfuric acid (H_2_SO_4_) (97%), and hydrogen peroxide (H_2_O_2_) (30% *w*/*w*) were purchased from Sigma-Aldrich (Hoboken, NJ, USA). The graphitic powder was acquired from Sharlau. The Fischer Scientific (Berlin, Germany) company provided the MB dye (purity 98%). Distilled water was obtained from the local water purification system used during the experiments. 

### 4.2. Synthesis of Graphene Oxide (GO) and FeWO_4_

Graphene oxide was prepared using the modified Hummer method, as reported in our previous work [23]. 

### 4.3. Synthesis of Iron Tungstate/Graphene Oxide (FeWO_4_/GO)

For the synthesis of the FeWO_4_/GO (FWGO) composite, the co-precipitation-assisted hydrothermal method was used. The sonication method was used to dissolve nearly 0.09 g of iron tungstate into 240 mL of deionized water. Sonication was accomplished by dissolving roughly 0.18 g of graphene oxide in 50 mL of deionized water (DI). The solutions were then vigorously magnetically stirred at room temperature for 30 min. The mixture was placed in an autoclave and heated at 160 °C for six hours. The final solution was cooled at room temperature (25 °C) and washed with water and ethanol. The samples were dried at 80 °C for 12 h in an oven.

### 4.4. Synthesis of Iodine-Doped Iron Tungstate (I-FeWO_4_)

I-FeWO_4_ was synthesized using a process described in our prior study. First, 1.176 g of Mohr’s salt was dissolved in 30 mL of deionized water. In this Mohr’s salt solution, we dissolved 0.99 g of sodium tungstate dihydrate and 1% potassium iodide in 30 mL of deionized water. For 30 min, magnetic stirring was employed to mix the precursors homogeneously. A 100 mL autoclave was filled to the top with the prepared solution and placed in the oven for 12 h at 200 °C. The autoclave was allowed to cool down to room temperature (25 °C), and the final solution was washed with distilled H_2_O and ethanol to remove contaminants before drying for 12 h at 80 °C [24].

### 4.5. Synthesis of Iodine-Doped Iron Tungstate/Graphene Oxide (I-FeWO_4_/GO)

**Figure 12:**
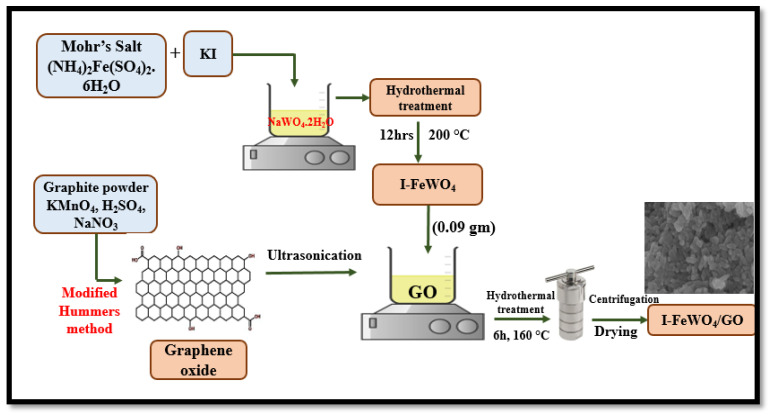
The schematic representation of I-FeWO_4_/GO.

A co-precipitation-assisted hydrothermal method was used to synthesize the novel I-FeWO_4_/GO (I-FWGO). For sonication, 0.09 g of iodine-doped iron tungstate was dissolved into 240 mL of deionized water. Similarly, about 0.18 g of graphene oxide was dispersed into 50 mL of deionized water, and the solutions were mixed using sonication at room temperature for 30 min. The solutions were poured into a 500 mL autoclave and placed into an oven at 160 °C for 6 h. Later, it was cooled down to room temperature and then washed with water and ethanol. Finally, the samples at 80 °C were dried for 12 h in the oven. The visual explanation of the synthesis process is shown in Figure 12.

### 4.6. Characterization and Equipment Details

The prepared novel composites FeWO_4_/GO and I-FeWO_4_/GO were characterized using X-ray diffraction (Philips PANalytical Xpert pro-DY 3805 powder XRD, Amsterdam, The Netherlands) for the phase analysis of the composites. Scanning electron microscopy equipped with energy-dispersive X-ray (SEM-EDX; FEI NOVA 450 NANOSEM, Austin, TX, USA) was used for the determination of surface morphologies and the elemental analysis of the samples. The functional groups were identified using FTIR (Agilent Technologies Cary 360 FTIR spectrophotometer, Santa Clara, CA, USA). The dye absorption was analyzed using a “CECIL CE-7200 UV–Vis spectrophotometer” (Hanover, Germany).

### 4.7. Photocatalytic Degradation Experiment

The photocatalytic degradation of MB under sunlight was observed to investigate the catalytic capacity of the novel I-FeWO_4_/GO catalyst generated. The support material has various applications in photocatalytic degradation due to its electrical and optical properties. Therefore, catalytic degradation is achieved by using materials similar to GO. To achieve adsorption–desorption equilibrium, the dye solution was put in the dark for half an hour. Following that, the dye solution with the catalyst was placed in sunlight for 120 min while using different oxidant dosages, catalyst concentrations, and pH levels. NaOH and HCl were used to maintain the pH of the solution. After each experiment, the catalyst was separated using a centrifuge machine, and the absorbance was assessed using a UV–Vis spectrophotometer at 665 nm. The percentage degradation of dye can be studied by using the following formula:

(7) $$\%\;\mathrm{Degradation}=\left(1-\frac{\mathrm{Co}}{\mathrm{Ct}}\right)\times 100\;$$

where Ct is the final absorbance after sunlight irradiation, and Co is the initial absorbance of the dyes. A solar power meter was used to determine the intensity of sun, and a light meter was utilized to measure brightness.

## 5. Conclusions

A co-precipitation-assisted hydrothermal approach was used to synthesize undoped and doped FeWO_4_/GO. Properties like morphology and band structure were used for evaluating the photocatalytic performance of the novel I-FeWO_4_/GO and FeWO_4_/GO. After the optimization of the various parameters, I-FeWO_4_/GO showed greater degradation efficiency than FeWO_4_/GO. Iodine doping restricts the grain boundary, increases the number of active sites for the adsorption of MB dye, and inhibits the recombination of holes and electrons. The crystalline sizes of FeWO_4_/GO and I-FeWO_4_/GO were 23.97 nm and 16.79 nm, respectively. I-FeWO_4_/GO and FeWO_4_/GO exhibited 97% and 92% of MB degradation after 120 min. The interaction and effects of the various parameters on degradation were studied by evaluating the results of RSM. Additionally, the novel I-FeWO_4_/GO also showed photocatalytic activity under UV. This research reveals that the electronic configuration of materials also improves the photocatalytic activity.

## Figures and Tables

**Table 1:** Reaction kinetics of FeWO_4_/GO and I-FeWO_4_/GO.

Photocatalyst	First-Order Kinetics		Second-Order Kinetics	
	R^2^	K_1_ (min^−1^)	R^2^	K_2_ (min^−1^)
FeWO_4_/GO	0.9848	0.016	0.8603	0.0019
I-FeWO_4_/GO	0.9903	0.0168	0.776	0.0026

**Table 2:** ANOVA table for I-FeWO_4_/GO.

Source	Sum of Squares	df	Mean Square	F Value	*p*-Value Prob > F	Remarks
Model	11,015.3	14	786.8	14,219.36	<0.0001	significant
A—pH	5.23	1	5.23	94.46	<0.0001	
B—Catalyst	14.42	1	14.42	260.51	<0.0001	
C—Oxidant	0.082	1	0.082	1.48	0.2432	
D—Time	261.36	1	261.36	4723.37	<0.0001	
AB	1004.89	1	1004.89	18,160.66	<0.0001	
AC	38.44	1	38.44	694.7	<0.0001	
AD	32.49	1	32.49	587.17	<0.0001	
BC	676	1	676	12,216.87	<0.0001	
BD	0.64	1	0.64	11.57	0.004	
CD	184.96	1	184.96	3342.65	<0.0001	
A2	4992.69	1	4992.69	90,229.29	<0.0001	
B2	5057.66	1	5057.66	91,403.45	<0.0001	
C2	336.8	1	336.8	6086.76	<0.0001	
D2	266.43	1	266.43	4815.01	<0.0001	
Residual	0.83	15	0.055			
Lack of Fit	0.53	10	0.053	0.88	0.5961	not significant
Pure Error	0.3	5	0.06			
Cor Total	11016.1	29				
SD.	0.24		R^2^		0.9998	
Mean	68.25		Adj. R^2^		0.9998	
C.V.	0.34		Pred. R^2^		0.9996	
PRESS	3.48		Adeq. Precision		335.872	

## Data Availability

Data is available on reasonable request.

## Sample Availability

Samples of the compounds are available from the authors.
